# 131. Impact of an Algorithm to Triage Patients Discharged from the Emergency Department with Blood Cultures Positive for *Staphylococcus aureus* or Coagulase-negative Staphylococci

**DOI:** 10.1093/ofid/ofad500.204

**Published:** 2023-11-27

**Authors:** Ethan Brenneman, Rebecca Theophanous, Jennifer Mando-Vandrick, Rachel Toler, Justin Jones, Elizabeth Keil, Grace Zhou, Hui-Jie Lee, Rebekah W Moehring, Rebekah Wrenn

**Affiliations:** Sturdy Memorial Hospital, Attleboro, Massachusetts; Duke University School of Medicine, Durham, North Carolina; Duke University Hospital, Durham, North Carolina; Duke Regional Hospital, Durham, North Carolina; Duke Raleigh Hospital, Raleigh, North Carolina; Duke University Hospital, Durham, North Carolina; Duke School of Medicine, Durham, North Carolina; Duke University Hospital, Durham, North Carolina; Duke University, Durham, NC; Duke University, Durham, NC

## Abstract

**Background:**

Patients may be discharged from the emergency department (ED) prior to final data from blood cultures. When positive, the ED provider must evaluate to determine next steps in caring for the patient. Up to 55% of positive blood cultures can be contaminants, and calling patients back to the ED may cause undue burden to the ED if no treatment or further evaluation is needed. We developed a toolkit to assist provider response to positive blood cultures for *Staphylococcus aureus* and Coagulase-negative staphylococci (CoNS) in patients discharged from the ED. It identifies which patients need to return to the ED (callback) and which do not (likely contaminant).

**Methods:**

We conducted a multi-center, retrospective cohort study of patients with positive blood cultures with staphylococci from January 2019 through October 2022 resulting after discharge from 3 EDs. The objective was to compare the rate of callback to the ED in a retrospective cohort to the rate guided by adhering to a newly developed callback algorithm. The rate of actual callback to ED and the rate of callback to ED based on adhering to the algorithm was calculated with 95% Wilson score binomial confidence intervals (CIs). Asymptotic McNemar’s test was used to compare the paired binomial proportions, and the difference in the rates was estimated with 95% CI using the Newcombe square-and-add approach.

**Results:**

Out of 188 patients discharged from the ED with the growth of staphylococci in the blood cultures, the rate of patient callback was 61.2% (115/188, 95% CI 55.2% to 66.8%), compared to the algorithm advised callback rate of 45.7% (86/188, 95% CI 39.9% to 51.7%). The difference between the actual callback and the algorithm-based callback rates was 15.4% (95% CI 9.8% to 20.9%, p< 0.001).

**Conclusion:**

Implementation of an algorithm-based response to blood cultures can reduce unnecessary callbacks to the ED without compromising patient safety.

**Disclosures:**

**Rebekah W. Moehring, MD, MPH, FIDSA, FSHEA**, UpToDate, Inc.: Author Royalties

